# The Treatment of Chronic Infections of the Nasopharynx with Benzoyl–Acetyl Peroxide

**Published:** 1906-09

**Authors:** John C. Warbrick

**Affiliations:** Chicago. Ill.; Former Instructor in Medicine, College of Physicians and Surgeons, The Medical Department of the University of Illinois


### The Treatment of Chronic Infections of the Nasopharynx with Benzoyl--Acetyl Peroxide.

BY JOHN C. WARBRICK, M. D., Chicago. Ill.

Former Instructor in Medicine, College of Physicians and Surgeons, The Medical Department of the University of Illinois.

I SEE a great many cases of chronic infections of the nose and nasopharynx in my practice, constituting pathological conditions characterised by hypersecretion, congested, sensitive mucosa and tendency to coryza; conditions resulting in hypertrophy of the turbinates, deflections of the septum, reflex irritations and a decided lessening of the general tone.

As the conditions described are unquestionably due to passive congestion superinduced by the presence upon the nasopharyngeal mucosa of pathogenic microorganisms, it is clear that what is wanted is a germicide which, while capable of destroying the germs, is not harmful to tissue, as so many of the efficient germicidal substances are. At various times, I have noticed in our literature mention of the use in nose and throat work of the benzoyl-acetyl peroxide (acetozone) first described in the American Chemical Journal, by Novy and Freer, of the University of Michigan. I thought that I would look into the adaptability of this substance to my work and my experiments proved so satisfactory that I have since made use of it almost as a routine measure.

I am satisfied, from my experiences, that acetozone solutions have a distinctly beneficial action in such cases as I shall describe further on. I have used benzoyl-acetyl-peroxide (acetozone) in aqueous solution containing a grain and a half of pure acetozone to six ounces of water, sprayed into the nose by an atomizer; but in the nose and mouth where more prolonged contact is desired than is possible to secure by aqueous solutions, I have utilized an oily solution which holds the germ-killing acetozone in place, adherent to the mucosa.

A satisfactory solution can now be obtained at the chemists under the name of acetozone inhalant, which answers every purpose of the laryngologist. Acetozone inhalent contains one per cent. of acetozone and five-tenths per cent. of chloretone in colorless liquid petrolatum. It possesses the characteristic properties of acetozone—germicidal and innocuous,—as demonstrated by repeated experiments. It is ready for instant use, is practically nonirritating and easily applied, the only instrument required being an oilatomizer.

In the following paragraphs I briefly describe my method of treating such cases. My patients report daily at the office, and as their progress toward recovery goes on, they come every other day, and later need to report only once or twice a week.

In the treatment of nasal catarrh of any character, the nasopharynx should first be cleansed of all accumulated secretion by the use of an alkaline fluid, such as Dobell's solution, or alkathymol, or some such mild solvent of the tenacious and infected mucus. My object is to reduce the irritability of the nasal mucous membrane and to protect it from the various irritants or infective material which produce hydrorrhea, sneezing, etc., as well as to render the mucosa an unsatisfactory culture field. I then spray into the nares a liberal quantity of the acetozone inhalant, the excess being removed by the towel or the patient's handkerchief. Of course, appropriate treatment of the general condition is always directed,—laxatives are given, diet and personal regime carefully attended to.

The following notes demonstrate the efficiency of the treatment and the class of cases to which I have applied it:

Case I. Mrs. F. K., age thirty-three years, by occupation a stenographer, came to me on January 27th, 1904. She stated that she had had catarrh for ten years and that she suffered from hay fever during the season. She complained of profuse discharge from the nares and some accumulation in the nasopharynx; also of a disagreeable taste in the mouth, and stomachic disturbance. She coughed in the morning, raising quite a quantity of phlegmatic expectoration. The face was sallow. In the left naris there was a small spur on the lower and anterior portion of the septum. Both nares were nearly filled with a thick serous exudate. The vocal cords were congested, as was also the pharyngeal wall. This patient received fifteen treatments, after which she was so much improved that she ceased her visits and treatments.

Case II. Mr. C. A. K., age thirty-five years, visited me on March 13, 1905, stating that his nose and throat had given him trouble for a long time; that there was a continual mucous discharge from the nasopharynx and that he expectorated a great deal of "phlegm" all the time, but this was aggravated in the morning. Had tried various treatments without avail. He smoked three or four cigars daily. The nasal mucous membrane was red and congested and there was a profuse stringy discharge accumulating on the posterior pharyngeal wall. He required ten treatments locally with the regularly prescribed regime to restore his normal condition.

Case III. Mrs G. A., aged fifty-eight years, housewife, came to me on August 11, 1905, with a history of having had catarrh for thirty years; an attack of ulcerative pharyngitis, for which she used a gargle, a long time ago; several attacks of bronchitis and one of pneumonia. She was always more or less hoarse and could not sing at all. She also complained of vertical and frontal headaches and pain across the eyes, which were aggravated on rainy days. Sense of smell had been lost for a year. She had a bad taste in her mouth and her stomach was out of order, flatulency, irregular bowels, with occasional crampy pains and a disordered liver. Previous weight, 165 pounds; at that time, 137 pounds.

The nasal mucous membrane was sensitive and there was considerable discharge from the anterior nares and an accumulation of mucus in the nasopharynx. Heart normal, liver slightly enlarged.

The patient had received treatment for years without any benefit to her nose and throat. She received twenty-three treatments, both local and general, and at the time of her dismissal was greatly improved.

